# CaMKII-δ9 Induces Cardiomyocyte Death to Promote Cardiomyopathy and Heart Failure

**DOI:** 10.3389/fcvm.2021.820416

**Published:** 2022-01-20

**Authors:** Mao Zhang, Junxia Zhang, Wenjia Zhang, Qingmei Hu, Li Jin, Peng Xie, Wen Zheng, Haibao Shang, Yan Zhang

**Affiliations:** ^1^State Key Laboratory of Membrane Biology, Institute of Molecular Medicine, College of Future Technology, Peking University, Beijing, China; ^2^Stanford Cardiovascular Institute, Stanford University School of Medicine, Stanford, CA, United States; ^3^Key Laboratory of Molecular Cardiovascular Sciences, School of Basic Medical Sciences, Institute of Cardiovascular Sciences, Ministry of Education, Peking University Health Science Center, Beijing, China; ^4^Beijing Key Laboratory of Cardiovascular Receptors Research, Beijing, China

**Keywords:** CaMKII, CaMKII-δ9, cardiomyocyte death, cardiomyopathy, heart failure, hypertrophy

## Abstract

Heart failure is a syndrome in which the heart cannot pump enough blood to meet the body's needs, resulting from impaired ventricular filling or ejection of blood. Heart failure is still a global public health problem and remains a substantial unmet medical need. Therefore, it is crucial to identify new therapeutic targets for heart failure. Ca^2+^/calmodulin-dependent kinase II (CaMKII) is a serine/threonine protein kinase that modulates various cardiac diseases. CaMKII-δ9 is the most abundant CaMKII-δ splice variant in the human heart and acts as a central mediator of DNA damage and cell death in cardiomyocytes. Here, we proved that CaMKII-δ9 mediated cardiomyocyte death promotes cardiomyopathy and heart failure. However, CaMKII-δ9 did not directly regulate cardiac hypertrophy. Furthermore, we also showed that CaMKII-δ9 induced cell death in adult cardiomyocytes through impairing the UBE2T/DNA repair signaling. Finally, we demonstrated no gender difference in the expression of CaMKII-δ9 in the hearts, together with its related cardiac pathology. These findings deepen our understanding of the role of CaMKII-δ9 in cardiac pathology and provide new insights into the mechanisms and therapy of heart failure.

## Introduction

Heart failure is a complex and heterogeneous syndrome resulting from impairment of ventricular filling or ejection of blood associated with symptoms of dyspnea, fatigue, as well as peripheral and/or pulmonary edema. Heart failure is one of the most prominent causes of hospitalization globally, with 3.6 million newly diagnosed patients annually imposing an unprecedented cost burden on the health care system (1, 2). The pathophysiological mechanisms of heart failure consist of cardiac injuries at multiple levels, including the myocardium, vasculature, pericardium, heart valves, electrical system, or a combination of cardiac abnormalities, among which cardiomyocyte death and hypertrophy are two critical factors.

Cardiomyocyte death significantly contributes to the progression of heart failure (3, 4). Multiple myocardial injury insults lead to cardiomyocyte death. Adult mammalian cardiomyocytes are terminally differentiated cells and have a minimal capacity for self-replacement. The loss of mammalian cardiomyocytes cannot be replenished from living cells, resulting in compromised cardiac function and heart failure. On the other hand, in response to myocardial injury or chronically increased hemodynamic load, cardiac mass increases due to cardiomyocyte hypertrophy to help maintain ejection performance. However, continued hemodynamic overload leads to the dilation of the heart and the thinning of the cavity walls, resulting in the change of myocardial geometry, an increase of wall stress, and cardiac dysfunction (5, 6).

Cardiomyocyte death and hypertrophy interact with each other and synergistically promote the progression of heart failure. Sustained cardiac pathological stresses result in progressive myocardial hypertrophy that eventually exceeds the capacity of the coronary vasculature to adequately perfuse the myocardial mass, leading to multiple foci of myocardial ischemia, cardiomyocyte death, myocardial fibrosis, and the deterioration of cardiac dysfunction. On the other hand, multiple myocardial injury insults cause cardiomyocyte death, and the surviving myocytes compensate by becoming hypertrophic to maintain normal cardiac function. Despite extensive efforts of evidence-based pharmacologic and device therapies, an unacceptable number of patients suffer impaired functional capacity, poor quality of life, and early death due to heart failure. Furthermore, hospitalized heart failure patients continue to experience unacceptably high post-discharge mortality and readmission rates, which have not been improved in the last two decades (7, 8). Therefore, it is of great importance to identify new therapeutic targets of heart failure.

Ca^2+^/calmodulin-dependent kinase II (CaMKII) is a serine/threonine protein kinase that modulates various biological functions and pathological processes in the heart (9–11). Excessive CaMKII activation is critically involved in multiple cardiac pathological conditions, such as myocardial ischemic injury (12–15), arrhythmia (16, 17), cardiac hypertrophy and remodeling (18, 19), and cardiomyopathy and heart failure (15, 19), and inhibition of CaMKII over-activation profoundly alleviates these cardiac diseases in animal models (13–15, 19–23). In cardiomyocytes, CaMKII plays a central role in regulating cell survival (12, 24, 25) and hypertrophy (18, 26, 27). CaMKII is encoded by four genes, CaMKII-α, β, γ, and δ, and CaMKII-δ is predominantly expressed in the heart. CaMKII-δ is alternatively spliced to generate 11 different variants (28–30). Different isoforms and splice variants possess distinct or even opposite biological and pathological functions (25, 31–35). Our recent study shows that CaMKII-δ9 is the most abundant CaMKII-δ splice variant in the human heart and acts as a central mediator of DNA damage and cell death in cardiomyocytes (35). The cardiac-specific CaMKII-δ9 transgenic mice develop extensive cardiomyopathy and heart failure. But the role of CaMKII-δ9 in cardiac physiology and pathology, especially its function in myocardial hypertrophy and heart failure, remains far from clear.

Here, we proved that CaMKII-δ9 mediates cardiomyocyte death instead of hypertrophy to elicit cardiomyopathy and heart failure. Thus, this study not only deepens our understanding of the role of CaMKII-δ9 in cardiac pathology, but also provides new insights of the mechanisms of heart failure.

## Materials and Methods

### Animals

Animals were maintained in the Center for Experimental Animals (an Association for Assessment and Accreditation of Laboratory Animal Care-accredited experimental animal facility) at Peking University, Beijing, China. The animals were randomly allocated to experimental groups. Both males and females were used. No non-inclusion or exclusion parameters were used in our studies. Investigators were not blinded to treatments, but no subjective assessments were made. All procedures involving experimental animals (mice, rats, and rhesus monkeys) followed protocols approved by the Committee for Animal Research of Peking University and conformed to the Guide for the Care and Use of Laboratory Animals.

Adult C57BL/6 mice and Sprague-Dawley rats were from Vital River Laboratories, Beijing, China. Rhesus monkeys were from our in-house cohort as previously reported (36). The animals were euthanized by intravenous injection of an overdose of sodium pentobarbital, and the tissues were quickly frozen in liquid nitrogen for protein and total RNA extraction.

The cardiac-specific CaMKII-δ9 transgenic mice was generated as previously described (35).

### *In vivo* KN-93 Treatment

Five-week-old wild-type and CaMKII-δ9 mice received *ip*. injection of either KN-93 (10 μmol/kg; Millipore, 422711) or a comparable volume of saline every other day. The survival rates were recorded for seven weeks, and cardiac function was then assessed by echocardiography.

### *In vivo* z-VAD Treatment

Five-week-old wild-type and CaMKII-δ9 mice received *ip*. injection of either z-VAD (0.5 mg/kg; Sigma, V116) or a comparable volume of saline twice a week. The survival rates were recorded for seven weeks, and cardiac function was then assessed by echocardiography.

### Human Heart Samples

Normal human ventricular tissues were from the NIH NeuroBioBank at the University of Maryland, Baltimore, MD as previously described (35).

### Animal Surgery and Treatment

Transverse aortic constriction (TAC) was performed in 6-week-old male mice as described before (35). Mice were anesthetized under 3% isoflurane *via* intubation, the chest was opened, the aortic arch was visualized, and a 7-0 silk suture was passed under the arch between the innominate and left common carotid arteries. The suture was secured around both the aorta and a 28-gauge needle, the needle was removed, the chest was closed, and the mouse was extubated. Sham-surgery mice underwent an identical procedure except for the aortic ligation. Mice were provided buprenorphine *via ip*. injection during recovery.

### Caspase 3/7 Activity Analysis

According to the manufacturer's instructions, caspase 3/7 activity was measured with a kit from Promega (Cat#: G8091).

#### Echocardiography

Echocardiographic analysis using a Vevo2100 digital imaging system (Visual Sonics, Toronto, ON, Canada) was performed under 1% isoflurane at 6 and 10 weeks of age, with mid-ventricular M and B mode measurements acquired in the parasternal short-axis view at the level of the papillary muscles. Once the mice were acclimated to the procedures, images were stored digitally on a magnetic, optical disk for review and analysis. Measurements of the LV internal end-diastolic diameter (LVIDd) were taken at the apparent maximal LV diastolic dimension. In contrast, the LV internal end-systolic diameter (LVIDs) measurements were taken at the time of the most anterior systolic excursion of the posterior wall. LV ejection fraction (EF) was calculated by the cubic method: LVEF (%) = {(LVIDd)^3^ − (LVIDs)^3^}/(LVIDd)^3^ × 100, and LV fractional shortening (FS) was calculated by FS (%) = (LVIDd - LVIDs)/LVIDd × 100. The data were averaged from five cardiac cycles.

### Histological Analysis

Histological analysis of heart tissues was as previously described (12). The CardioTACSTM *in situ* apoptosis detection kit (Roche Applied Science, Cat#: 11684795910) was used for TUNEL staining as previously described (35). Immunohistochemistry was performed on heart tissues with anti-γH2AX antibody and the DNA damage levels were determined by the percentage of γH2AX positive cells.

### Gene Expression Analysis and Primers

The following primer pairs were used for quantitative real-time PCR:

**Table:** 

**Gene**	**Direction**	**Sequence 5'-3'**
18S	Forward	GGAAGGGCACCACCAGGAGT
18S	Reverse	TGCAGCCCCGGACATCTAAG
ANP	Forward	TTCTTCCTCGTCTTGGCCTTT
ANP	Reverse	GACCTCATCTTCTACCGGCATCT
BNP	Forward	AAGTCCTAGCCAGTCTCCAGA
BNP	Reverse	GAGCTGTCTCTGGGCCATTTC

Amplification was performed as follows: 95°C for 3 s and 40 cycles at 95°C for 15 s and 60°C for 30 s. Data are the average of at least three independent experiments.

### Isolation, Culture, and Adenoviral Infection of Ventricular Myocytes

Neonatal rat ventricle myocytes (NRVMs) were isolated from 1-day-old Sprague-Dawley rats, and adenovirus-mediated gene transfer was implemented using methods described previously (35). NRVMs were exposed to KN-93 (5 μM) or isopropanol (ISO,10 μM) treatment.

Adult rat ventricle myocytes (ARVMs) were isolated from the hearts of 2–3-month-old Sprague-Dawley rats using a standard enzymatic technique, then cultured and infected with adenoviral vectors at a multiplicity of infection (MOI) indicated as described previously (35). Briefly, myocytes were plated at a density of 0.5 to 1 × 10^4^/cm^2^ on coverslips or in dishes precoated with 10 μg/ml laminin. The culture medium was M199 (Sigma) plus 5 mmol/L creatine, 2 mmol/L l-carnitine, 5 mmol/L taurine, 0.1% insulin-transferrin-selenium-X, 1% penicillin and streptomycin, and 25 mmol/L HEPES, pH 7.4, at 37°C. Adenovirus-mediated gene transfer was implemented by adding adenoviral vectors (35) into the culture dish. The experiments were done with cells cultured 24 h after infection unless specified otherwise.

### Western Blot

Western blot was performed as previously described (12).

### RNA Interference-Mediated Gene Silencing

For gene-silencing assays, siRNAs with 19 nucleotides in length, carrying a dTdT overhang at the 3' terminus, were designed using the Invitrogen website. Cardiomyocytes were transfected with siRNA using Lipofectamine RNAiMAX (Invitrogen) following the manufacturer's instructions (25).

**Table:** 

**Gene**	**Sequence 5'-3'**
Scrambled	UUCUCCGAACGUGUCACGU
CaMKII-δ9	GCUACUGGGCAUCAUCAUA

### Materials

Antibodies against the following proteins were used: UBE2T for mouse [Aviva Systems Biology, ARP-43145 (Lot#: QC13585-40506; 1:1000)], t-CaMKII-δ [GeneTex, GTX111401 (Lot#: 40058; 1:1000)], γH2AX (Millipore, 05-636, clone JBW301 (Lot#: 2884537; 1:1000 for western blots, 1:200 for immunohistochemistry), Cleaved Caspase-3 [Cell Signaling Technology, 9661 (1:1000)] and GAPDH [EASYBIO, BE0023, clone 2B8 (1:10000)]. Antibody against Exon 16 of CaMKII-δ9 was generated as described (35). ISO, z-VAD, and Doxorubicin were from Sigma-Aldrich. KN-93 was from Millipore (Cat# 422711).

### Statistical Analysis

Data are expressed as mean ± S.E.M. Statistical analysis was performed with GraphPad Prism version 8.4 (GraphPad Software, Inc.). Data sets were tested for normality of distribution with the Kolmogorov-Smirnov test. Data groups (two groups) with normal distributions were compared using the two-sided unpaired Student's *t-*test. The Mann-Whitney *U*-test was used for nonparametric data. One-way or two-way ANOVA assessed comparisons between multiple groups with Bonferroni *post hoc* analysis. ^*^P < 0.05; ^**^P < 0.01; NS, not significant. No statistical method was used to predetermine sample size.

## Results

### CaMKII Inhibition Prevents CaMKII-δ9-Induced Cardiomyopathy and Heart Failure

**Figure 1:**
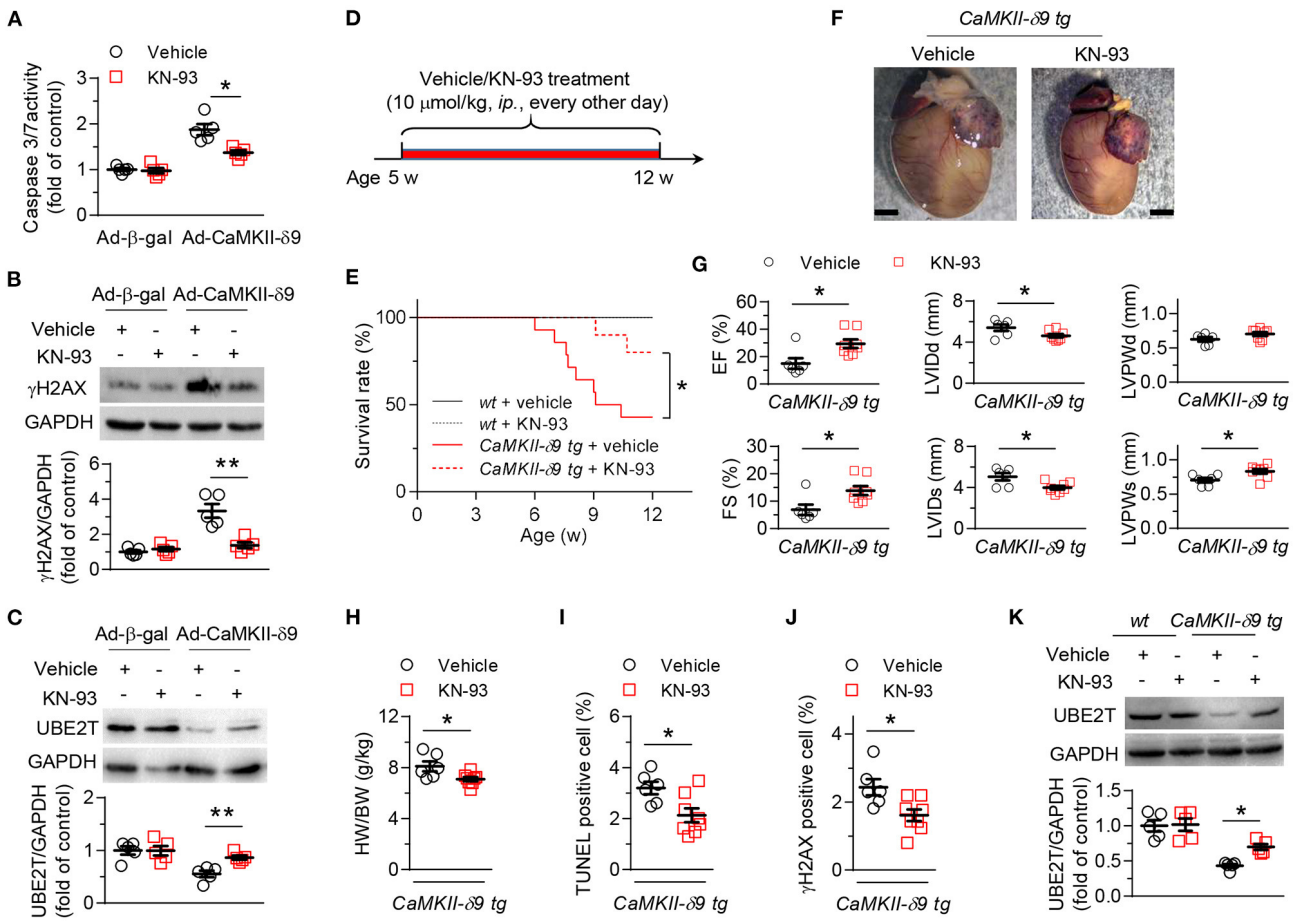
KN-93 treatment prevents CaMKII-δ9-induced cardiomyopathy and heart failure. **(A–C)**, Cell viability assayed by caspase 3/7 activity **(A)** and representative western blots and statistical data showing the levels of γH2AX **(B)** and UBE2T **(C)** in NRVMs infected with Ad-β-gal or Ad-CaMKII-δ9 with or without KN-93 treatment (5 μM) (*n* = 5). **(D)** Experimental protocol for *in vivo* KN-93 treatment in *CaMKII-*d*9 tg* mice. The mice were treated with KN-93 (10 μmol/kg, *i.p*.) every other day from 5 to 12 weeks of age. **(E)** Kaplan-Meier survival curves of *wt* and *CaMKII-*δ*9 tg* mice with or without KN-93 treatment (*n* = 8 for *wt* vehicle, *n* = 7 for *wt* KN-93, *n* = 14 for *CaMKII-*δ*9 tg* vehicle, *n* = 10 for *CaMKII-*δ*9 tg* KN-93). **(F–J)** Gross morphology of the hearts **(F)**, statistical data of the left ventricle echocardiography **(G)**, ratio of heart weight to body weight **(H)**, and statistical data of TUNEL **(I)** and γH2AX **(J)** staining of the hearts from *CaMKII-*δ*9 tg* mice with or without KN-93 treatment. EF, ejection fraction; FS, fractional shortening; LVIDd and LVIDs, diastolic and systolic left ventricular internal diameter; LVPWd and LVPWs, diastolic and systolic left ventricular posterior wall thickness. Scale bar, 2 mm (*n* = 6 for vehicle, *n* = 8 for KN-93). **(K)** Representative western blots and statistical data showing the levels of UBE2T from the hearts of *wt* and *CaMKII-*δ*9 tg* mice with or without KN-93 treatment (*n* = 5). Data are mean ± S.E.M. *P < 0.05, **P < 0.01; two-way ANOVA **(A–C,K)**, log-rank (Mantel-Cox) test, or Student's *t*-test **(E,G–J)**.

First, we investigated whether CaMKII kinase activity was required for CaMKII-δ9-induced cardiomyocyte death, cardiomyopathy, and heart failure. KN-93 is the classic CaMKII inhibitor, inhibiting CaMKII kinase activity (37) and ameliorating multiple cardiac diseases in experimental models (38). We first set up cardiomyocyte injury models with cultured NRVMs with CaMKII-δ9 overexpression and found that pretreatment of KN-93 (5 μM) alleviates the CaMKII-δ9-induced cardiomyocyte death as indexed by caspase 3/7 activity (Figure 1A). We have previously shown that CaMKII-δ9 induced DNA damage in cardiomyocytes through the degradation of UBE2T (35). Here, we proved that in NRVMs, KN-93 suppressed CaMKII-δ9-mediated UBE2T degradation and subsequent DNA damage (Figures 1B,C).

We have constructed the mice with cardiac-specific overexpression of CaMKII-δ9 (*CaMKII-*δ*9 tg* mice) (35). In *CaMKII-*δ*9 tg* mice, treatment with KN-93 (10 μmol/kg, *ip*., every other day) from the age of 5 weeks attenuates premature animal death, cardiac hypertrophy, myocardial dysfunction, and cardiomyocyte DNA damage and cell death (Figures 1D–K). Therefore, CaMKII kinase activity is required for CaMKII-δ9-induced cardiomyocyte death, cardiomyopathy, and heart failure.

### Inhibition of Cardiomyocyte Death Alleviates CaMKII-δ9-Induced Cardiomyopathy and Heart Failure

Based on the phenotypes of the *CaMKII-*δ*9 tg* mice, there are three possible functions of CaMKII-δ9 in the heart: First, it directly induces cardiomyocyte hypertrophy, which indirectly causes myocyte death when hypertrophy progresses to decompensation, or it directly induces cardiomyocyte death, and the surviving myocytes compensate by becoming hypertrophic, or both.

**Figure 2:**
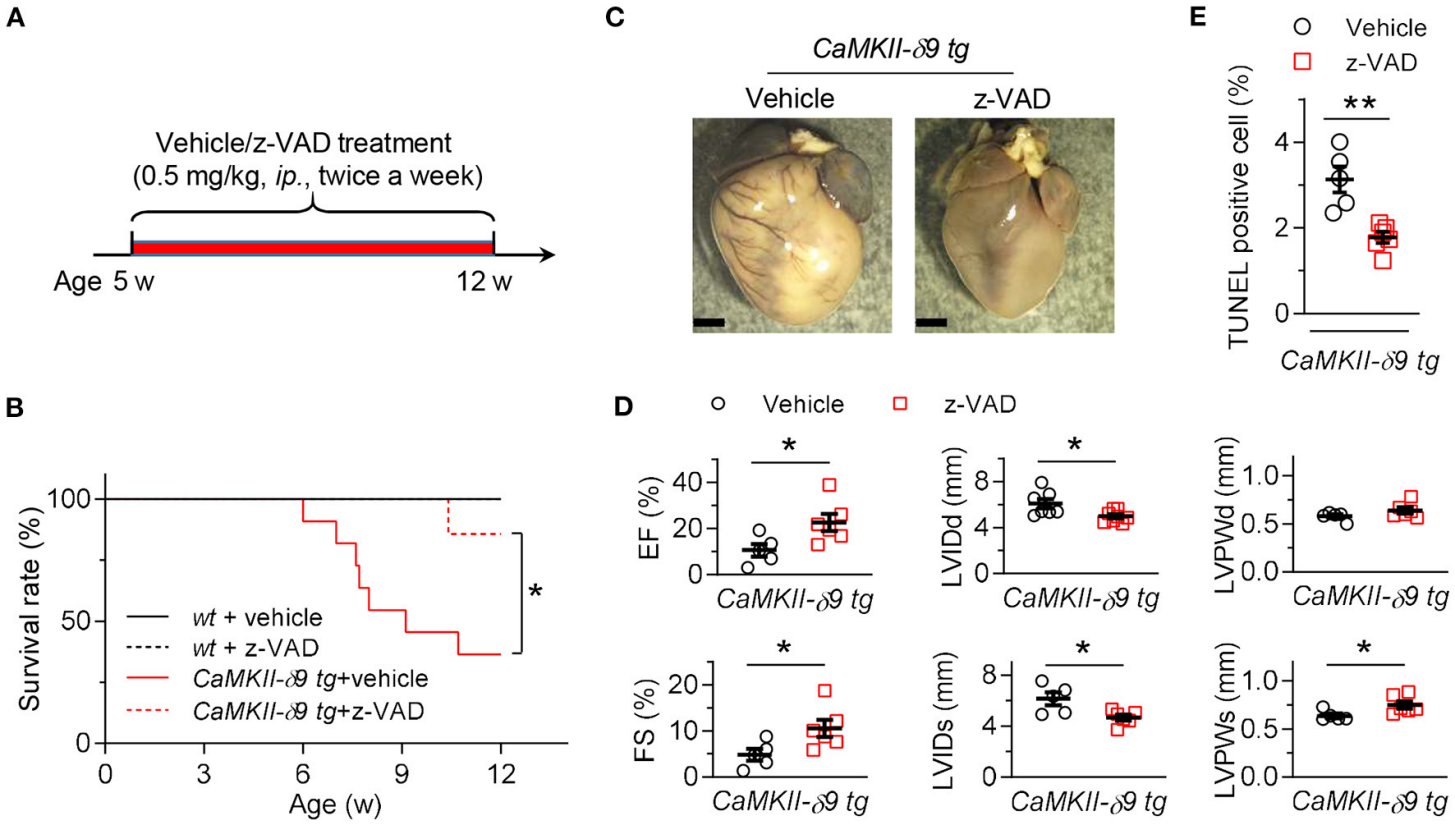
Inhibition of cardiomyocyte death alleviates CaMKII-δ9-induced cardiomyopathy and heart failure. **(A)** Experimental protocol for *in vivo* z-VAD treatment in *CaMKII-*δ*9 tg* mice. The mice were treated with z-VAD (0.5 mg/kg, *i.p*.) twice a week from 5 to 12 weeks of age. **(B)** Kaplan-Meier survival curves of *wt* and *CaMKII-*δ*9 tg* mice with or without z-VAD treatment (*n* = 6 for *wt* vehicle, *n* = 5 for *wt* z-VAD, *n* = 11 for *CaMKII-*δ*9 tg* vehicle, *n* = 7 for *CaMKII-*δ*9 tg* z-VAD). **(C–E)** Gross morphology of the hearts **(C)**, statistical data of the left ventricle echocardiography **(D)**, and statistical data of TUNEL staining of the hearts **(E)** from *CaMKII-*δ*9 tg* mice with or without z-VAD treatment. Scale bar, 2 mm (*n* = 5 for vehicle, *n* = 6 for z-VAD). Data are mean ± S.E.M. NS, not significant; *P < 0.05, **P < 0.01; log-rank (Mantel-Cox) test **(B)**, or Student's *t*-test **(D,E)**.

To distinguish these possibilities, we first treated the *CaMKII-*δ*9 tg* mice with z-VAD (5 mg/kg, *ip*., twice a week), a caspase inhibitor, from the age of 5 weeks to inhibit cardiomyocyte death (Figure 2A). Our data showed that the premature animal death in *CaMKII-*δ*9 tg* mice was markedly attenuated by z-VAD treatment (Figure 2B). Furthermore, cardiomyocyte death, cardiac hypertrophy, and heart dysfunction in *CaMKII-*δ*9 tg* mice were also ameliorated by z-VAD (Figures 2C–E), indicating that the cytosolic execution of apoptosis contributes to CaMKII-δ9-induced cardiomyocyte death, and cardiomyocyte death is essential for CaMKII-δ9-induced cardiomyocyte death, cardiomyopathy, and heart failure.

### CaMKII-δ9 Does Not Directly Regulate Cardiac Hypertrophy

**Figure 3:**
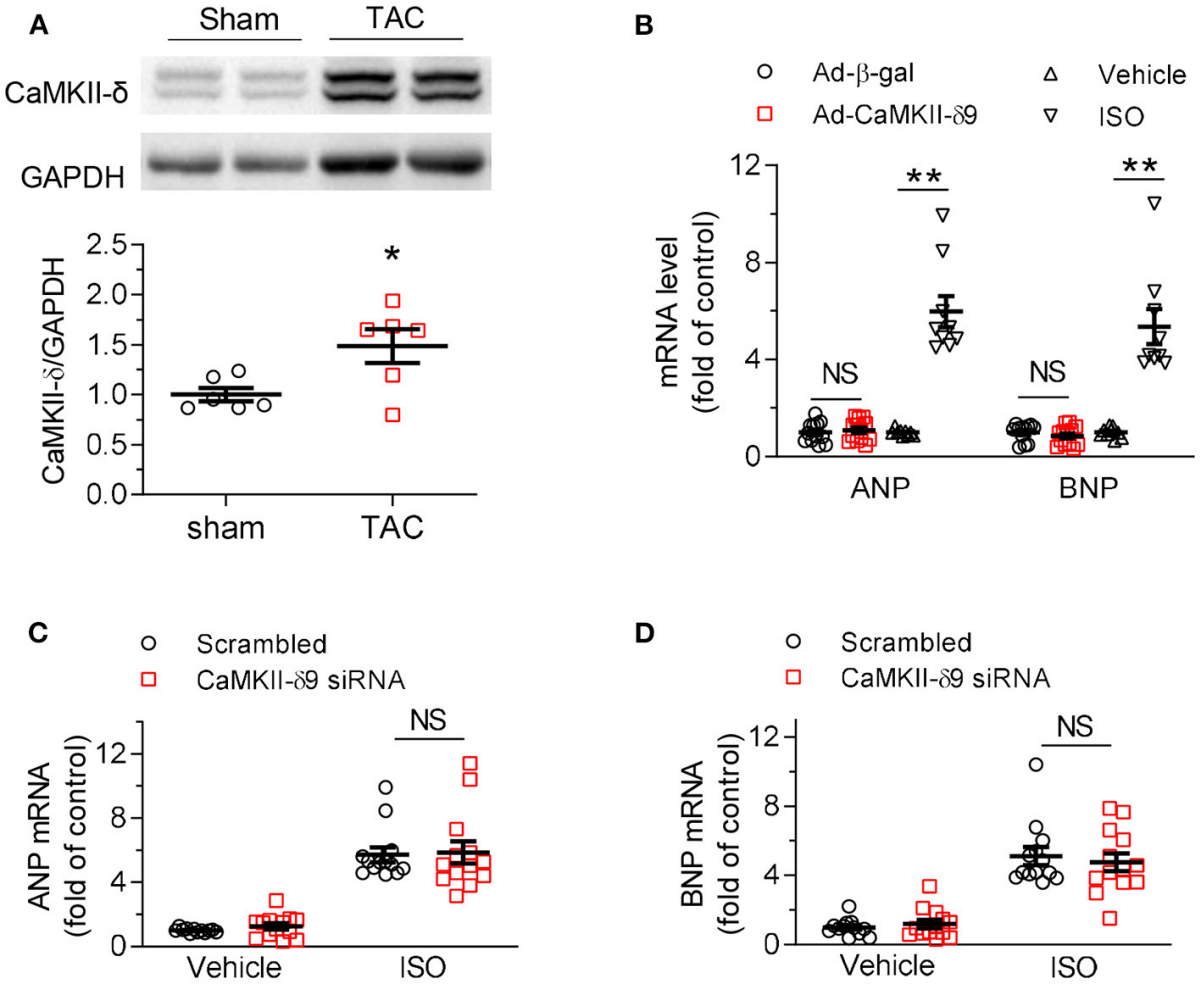
CaMKII-δ9 does not directly regulate cardiac hypertrophy. **(A)** Representative western blots and statistical data showing the levels of CaMKII-δ in the hearts of the mice 2 weeks after sham or transverse aortic constriction (TAC) surgery. *n* = 6. **(B)** Averaged data of mRNA levels of ANP and BNP assayed by real-time PCR in NRVMs infected with Ad-β-gal or Ad-CaMKII-δ9 (50 MOI, 48 h; Ad-β-gal, *n* = 12; Ad-CaMKII-δ9, *n* = 13). The NRVMs treated with isoproterenol (ISO, 10 mM, 24 h) were used as positive control (*n* = 9). **(C,D)** Averaged data of mRNA levels of ANP **(C)** and BNP **(D)** assayed by real-time PCR in NRVMs transfected with scrambled or CaMKII-δ9 siRNA with or without ISO treatment (10 μM, 24 h; Vehicle scrambled, *n* = 12; other groups, *n* = 13). Data are mean ± S.E.M. *P < 0.05, **P < 0.01; NS, not significant; Student's *t*-test **(A,B)**, or two-way ANOVA **(C,D)**.

We next investigated the role of CaMKII-δ9 in cardiomyocyte hypertrophy. In the mouse hearts two weeks after sham or TAC surgery, CaMKII-δ protein abundance was markedly increased (Figure 3A). To avoid complex *in vivo* compensations, we used cultured NRVMs in conjunction with adenoviral gene transfer. We found that although total CaMKII-δ protein was upregulated in hypertrophic hearts, overexpression of CaMKII-δ9 did not alter the expression of cardiac hypertrophic genes, including atrial natriuretic peptide (ANP) and brain natriuretic peptide (BNP). In contrast, as a positive control, isoproterenol treatment profoundly increased their expression (Figure 3B). Moreover, the knockdown of CaMKII-δ9 did not alter the hypertrophy phenotype in NRVM treated with isopropanol (Figures 3C,D). These data indicate that cardiac CaMKII-δ9 is not directly involved in cardiomyocyte hypertrophy. The myocardial hypertrophy in *CaMKII-*δ*9 tg* mice is the compensatory response of the surviving cardiomyocyte to maintain cardiac function.

### CaMKII-δ9 Downregulates UBE2T/DNA Repair Signaling to Induce Adult Cardiomyocyte Death

Our previous study has shown that CaMKII-δ9 binds to ubiquitin-conjugating enzyme E2T (UBE2T) to promote its phosphorylation and degradation, disrupting UBE2T-dependent DNA repair and leading to the accumulation of DNA damage and genome instability, which results in cardiomyocyte death (35). However, the study was performed in the context of immature cardiomyocytes, including NRVMs and embryonic stem cell-derived cardiomyocytes. Given the differences between immature and mature cardiomyocytes in terms of morphology, gene expression, and proliferation capacity, here, we investigated the role of CaMKII-δ9 in adult cardiomyocytes, together with the underlying mechanisms.

**Figure 4:**
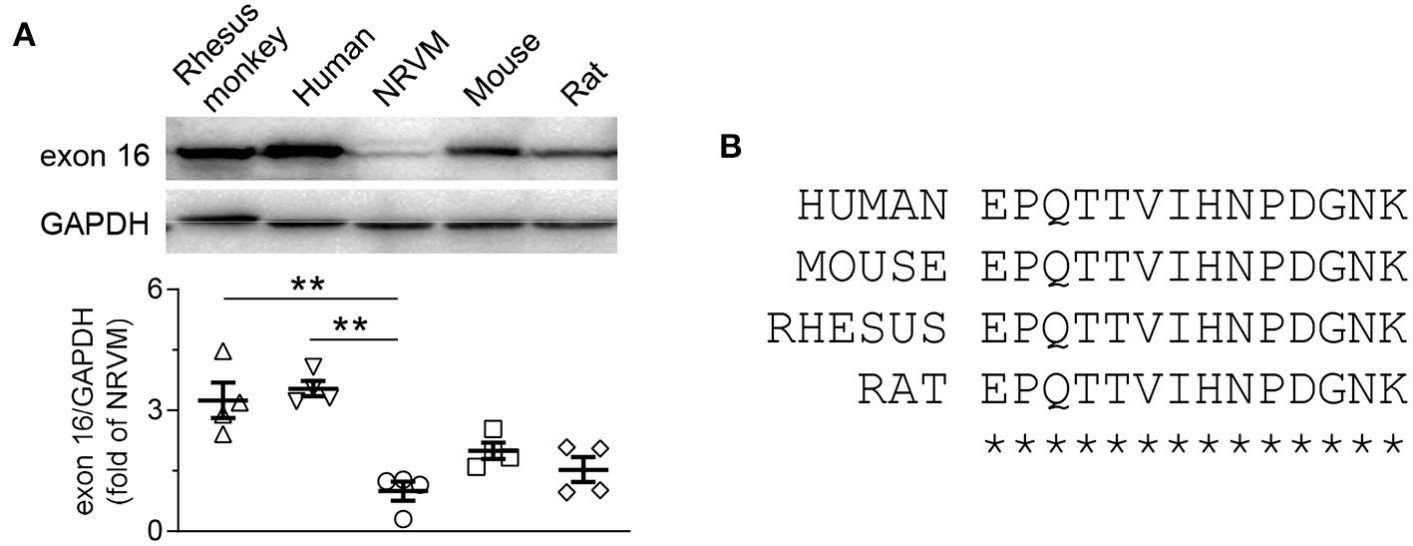
CaMKII-δ9 is abundant in the adult hearts of different species. **(A)** Representative western blots and statistical data showing the levels of CaMKII-δ9 in the hearts of rhesus monkey and human, neonatal rat ventricular myocytes (NRVMs), adult mouse together with rat (*n* = 4). Data are mean ± S.E.M. **P < 0.01; one-way ANOVA. **(B)** Amino acid sequences of the peptides encoded by the exon 16 of CaMKII-δ of human, mouse, rhesus monkey and rat.

First, we compared the protein abundance of CaMKII-δ9 in NRVMs with the adult hearts of multiple species, including human, monkey, rat, and mice (Figure 4A). Since these four species share the same amino-acid sequence encoded by exon 16 of CaMKII-δ (Figure 4B), the anti-exon 16 antibodies would be expected to work equally in these species. The protein abundance of CaMKII-δ9 in the heart of human, monkey, mice, and rat (adult) was 3.54 ± 0.19, 3.25 ± 0.44, 2.00 ± 0.20 and 1.53 ± 0.31-fold of that in NRVMs, respectively (Figure 4A), suggesting that the levels of CaMKII-δ9 are higher in adult hearts.

**Figure 5:**
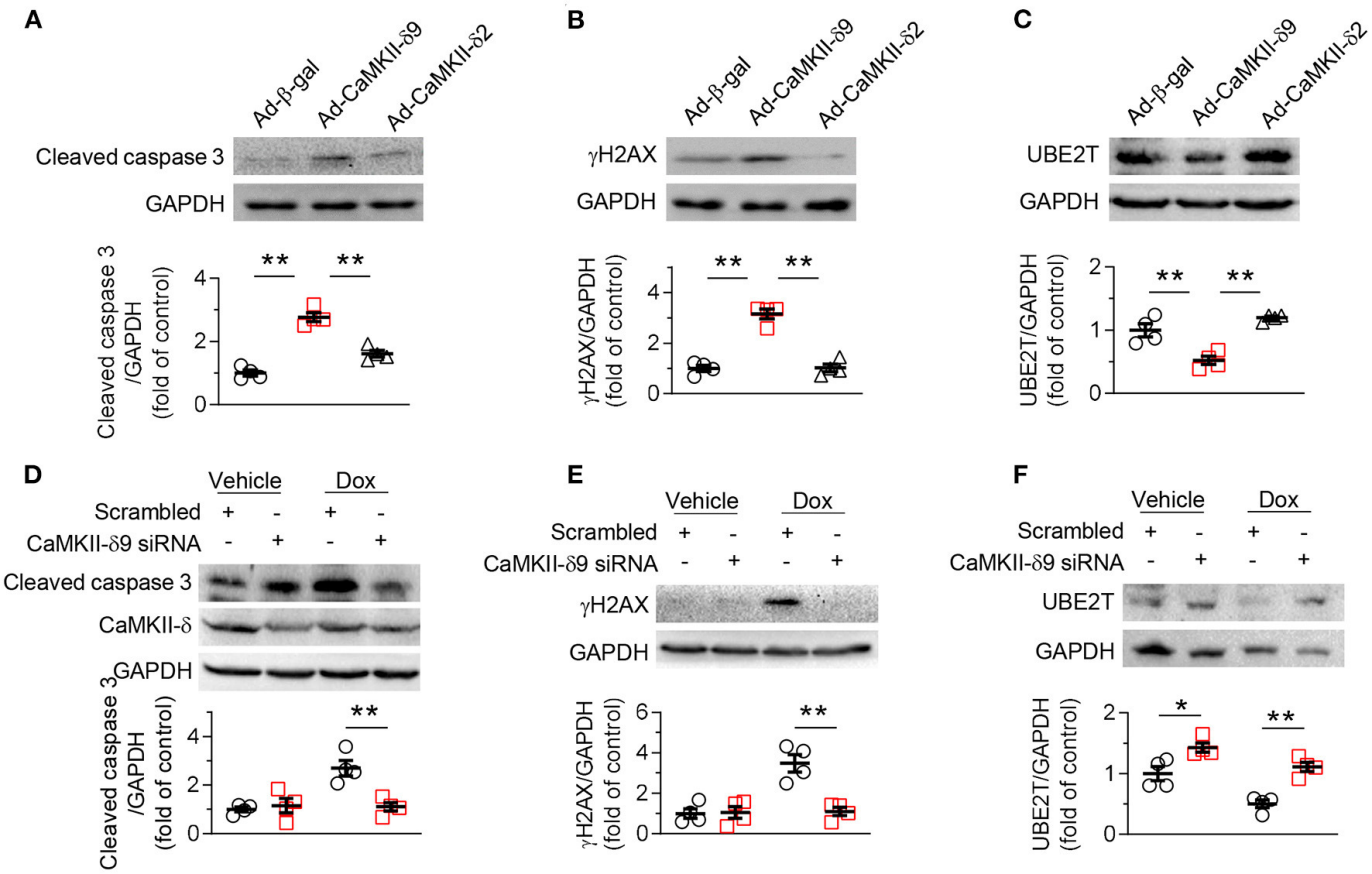
CaMKII-δ9 mediates DNA damage and cell death in adult cardiomyocytes. **(A–C)** Representative western blots and statistical data showing the levels of cleaved caspase 3 **(A)** γH2AX **(B)** and UBE2T **(C)** in adult rat ventricular myocytes (ARVMs) infected with Ad-β-gal, Ad-CaMKII-δ9, or Ad-CaMKII-δ2 (100 MOI, 24 h) (*n* = 4). **(D–F)** Representative western blots and statistical data showing the levels of cleaved caspase 3 **(D)**, γH2AX **(E)** and UBE2T **(F)** in ARVMs infected with scrambled or CaMKII-δ9 siRNA with or without Dox treatment (1 mM, 16 h) (*n* = 4). Data are mean ± S.E.M. *P < 0.05, **P < 0.01; one-way ANOVA **(A–C)** or two-way ANOVA **(D–F)**.

Functionally, our data indicated that similar to the findings in NRVMs, at a comparable expression level, CaMKII-δ9 elicited much more severe cell death, DNA damage, and UBE2T degradation than CaMKII-δ2 in ARVMs (Figures 5A–C). Consistently, knockdown of CaMKII-δ9 with its specific siRNA prevented doxorubicin-induced cell death, DNA damage, and UBE2T degradation in ARVMs (Figures 5D–F). These data indicate that enhanced CaMKII-δ9 activation triggers the death of both adult and neonatal cardiomyocytes by suppressing UBE2T-dependent DNA repair signaling. Therefore, although CaMKII-δ9 differs in protein abundance between neonatal and adult cardiomyocytes, its function and downstream signaling are the same.

### There Is No Gender Difference in CaMKII-δ9-Induced Cardiac Pathology

Previous studies have shown significant differences in the role of CaMKII-δ2 and -δ3 in males and females, and CaMKII activation is not necessarily deleterious in female cardiopathology (39). Thus, in order to fully understand the clinical significance of CaMKII-δ9 in the prevention and therapy of heart failure, we compared the possible gender differences of CaMKII-δ9 in terms of its expression and function in animals.

**Figure 6:**
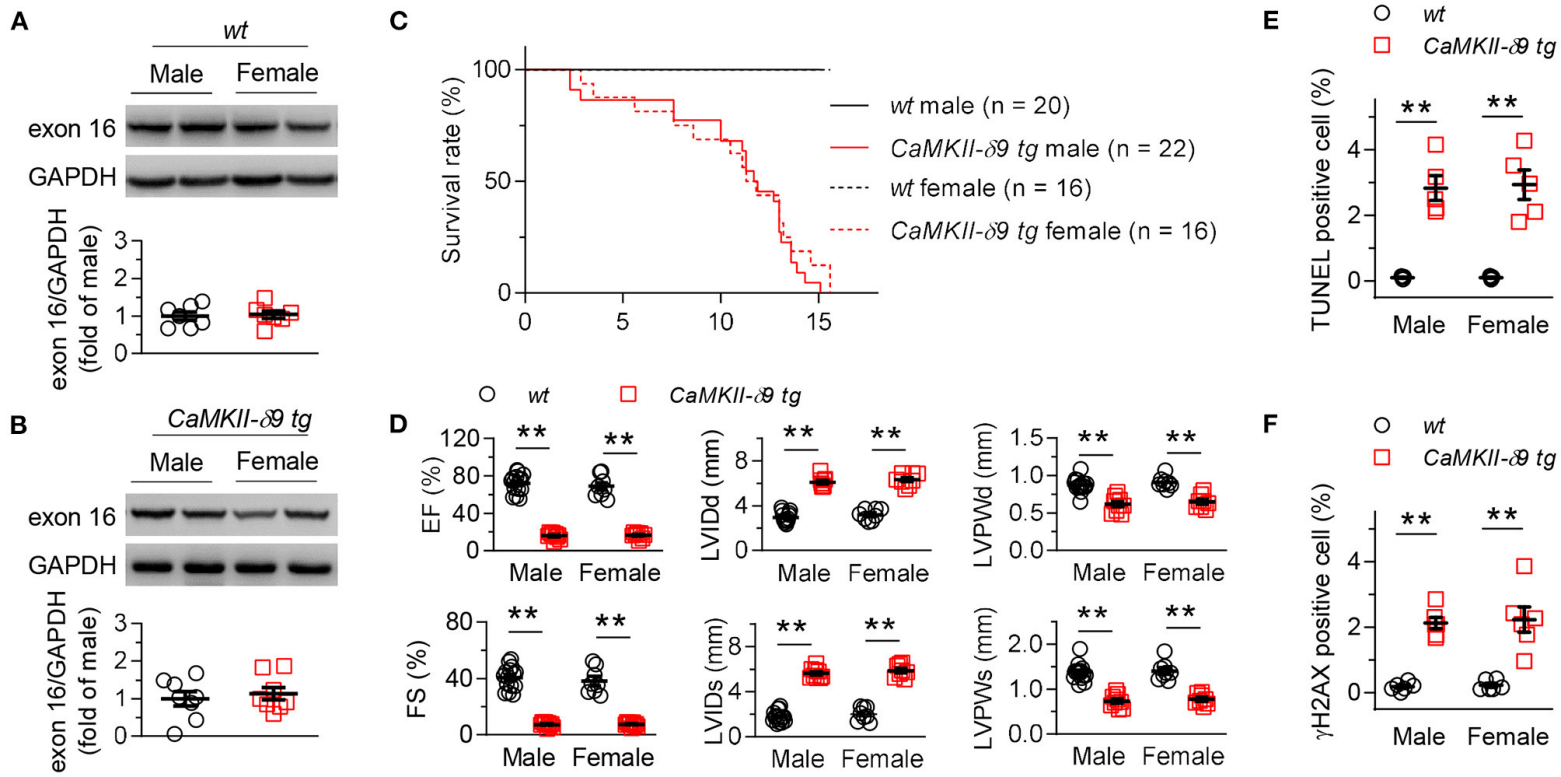
No gender difference in the CaMKII-δ9-induced cardiac pathology. **(A,B)** Representative western blots and statistical data showing the levels of CaMKII-δ9 from the hearts of *wt* (**A**, *n* = 7) and *CaMKII-*δ*9 tg* mice (**B**, *n* = 8). **(C)** Kaplan-Meier survival curves of male and female *wt* and *CaMKII-*δ*9 tg* mice (*n* = 20 for *wt* male, *n* = 22 for *CaMKII-*δ*9 tg* male, *n* = 16 for *wt* female and *CaMKII-*δ*9 tg* female). **(D)** Statistical data of the left ventricle echocardiography of male and female *wt* and *CaMKII-*δ*9 tg* mice at 10 weeks of age (*n* = 15 for *wt* male, *n* = 9 for *CaMKII-*δ*9 tg* male, *n* = 8 for *wt* female and *CaMKII-*δ*9 tg* female). EF, ejection fraction; FS, fractional shortening; LVIDd and LVIDs, diastolic and systolic left entricular internal diameter; LVPWd and LVPWs, diastolic and systolic left ventricular posterior wall thickness. **(E,F)** Statistical data of TUNEL (**E**, *n* = 5) and γH2AX (**F**, *n* = 6) staining of the hearts of male and female *wt* and *CaMKII-*δ*9 tg* mice at the age of 10 weeks (*n* = 5). Data are mean ± S.E.M. **P < 0.01; Student's *t*-test **(A,B)**, log-rank (Mantel-Cox) test **(C)**, or two-way ANOVA **(D–F)**.

We found that there was no gender difference of myocardial CaMKII-δ9 abundance in *wt* and *CaMKII-*δ*9 tg* mice (Figures 6A,B). In addition, animal viability, cardiac morphology and function, as well as cardiomyocyte apoptosis and DNA damage did not differ, either (Figures 6C–F). In this way, there is no gender difference in terms of the expression and function of CaMKII-δ9 in the mice hearts.

## Discussion

In the current study, we demonstrate the pathophysiological function of CaMKII-δ9 in the development of cardiomyopathy and heart failure, especially distinguished its role in hypertrophy and cardiomyocyte death. Specifically, CaMKII-δ9 mediates cardiomyocyte death, instead of hypertrophy, to promote the progression of cardiomyopathy and heart failure. In addition, we show that the function and the corresponding signaling pathway of CaMKII-δ9 are similar in immature and mature cardiomyocytes without gender difference.

Heart failure is a global public health problem and heavy finical burden to the patients. Despite significant efforts in the basic and clinical research to pursue the strategy of its prevention and therapy, it remains a huge unmet medical need. Cardiomyocyte death plays an essential role in the progression of cardiomyopathy and heart failure, and the inhibition of cardiomyocyte death alleviated heart failure (40–42). CaMKII is a key player in mediating cardiomyocyte death, and inhibition of CaMKII profoundly protects the cardiomyocyte against cell death induced by multiple pathological insults.

Further studies showed that different CaMKII-δ splice variants exert opposite functions in regulating cardiac cell viability. The cytosolic variant, CaMKII-δ2 (also named CaMKII-δC) facilities cardiomyocyte death, whereas the nuclear variant, CaMKII-δ3 (also named CaMKII-δB), is protective (25, 31–33). We recently provided evidence that CaMKII-δ9, instead of CaMKII-δ2, is the most critical cytosolic CaMKII variant in the human heart (35). Functionally, CaMKII-δ9 is more potent in inducing cardiomyocyte death than CaMKII-δ2 and plays an essential role in developing cardiomyopathy and heart failure (35). Here, we further demonstrate that CaMKII-δ9 directly elicits cardiomyocyte death, but not cardiac hypertrophy, to mediate the progression of heart failure. Thus, we showed a clear picture of the pathophysiological action of CaMKII-δ9, the major variant in human hearts, in the mediation of cardiomyopathy and heart failure, suggesting a new therapeutic strategy to target CaMKII-δ9 against human heart failure. Importantly, our data proved that CaMKII-δ9 downregulated UBE2T, impaired DNA repair machinery, and consequently elicited cardiomyocyte death and heart failure in the adult hearts of both male and female animals, which further enhances the clinical perspective of CaMKII-δ9 in the therapy of cardiac diseases.

During heart failure, CaMKII (in the human heart, mainly CaMKII-δ9) was activated by multiple cardiac pathological insults, including neurohumoral agonist signaling (43), oxidant stress (44–47), hyperglycemia (48, 49), ischemic injury (12, 13, 50–52), cardiac toxic drugs (12, 53), and other adverse stimuli associated with increased intracellular calcium levels (54, 55). The activated CaMKII mediates the phosphorylation of Ca^2+^ homeostatic proteins to enhance their activity and improve the performance of physiological events such as excitation-contraction coupling and fight/flight mechanical responses, which helps maintain normal cardiac function. However, excessive CaMKII activation caused by continuous myocardial stress promotes cardiac myocyte death and the deterioration of cardiomyopathy. We recently identified DNA damage as the specific downstream effector of CaMKII-δ9 in the induction of cardiac injury, and CaMKII-δ9 is much more potent in the induction of cardiomyocyte death than CaMKII-δ2 (35). In addition, some other mechanisms including inflammation (46, 50, 52, 56), mitochondrial stress (57), endoplasmic reticulum stress (47, 58–60), and p53 activation (61) have been shown to act as the downstream signaling of CaMKII-induced cardiomyocyte death. But compared with other splice variants, especially CaMKII-δ2, whether CaMKII-δ9 exerts similar functions in regulating these mechanisms still merits further investigation.

CaMKII has also been established to be a central mediator of cardiomyocyte hypertrophy. CaMKII is activated during cardiac hypertrophy, and inhibition of CaMKII profoundly alleviates myocardial hypertrophy, cardiomyopathy, and heart failure (22, 62, 63). Mechanically, CaMKII phosphorylates multiple substrates, including myocyte enhancer factor 2 (MEF2), histone deacetylases (HDACs), and histone H3, to induce a hypertrophic transcriptional response in cardiomyocytes (64–66). But all the previous studies are based on CaMKII-δ2 and CaMKII-δ3. Here in our study, we showed that different from the other variants, CaMKII-δ9 did not directly increase the expression levels of the hypertrophic genes in cardiomyocytes, and inhibition of CaMKII-δ9 failed to block isoproterenol-induced cardiomyocyte hypertrophy, implicating that CaMKII-δ9 is not involved in the regulation of cardiac hypertrophic response. In human cardiomyocytes, CaMKII-δ3 and CaMKII-δ9 are the major CaMKII splice variants, localized in the nuclei and cytosol, respectively. Our current data combined with the previous studies suggest that there is functional specialization of these variants in addition to the distinct subcellular localization. CaMKII-δ9 is responsible for regulating calcium handling and contraction and induces cardiomyocyte death; on the other hand, CaMKII-δ3 mediates the hypertrophic gene expression and protects against cardiac cell death. Therefore, the specific intervention of CaMKII-δ splice variant to target specific cardiac physiological and pathological functions is a promising strategy to improve the therapy of heart failure and other cardiac diseases.

In addition to ischemic heart diseases, heart failure can be caused by many other diseases and pathological conditions, including hypertension, diabetes, and anti-cancer drugs. Since CaMKII has been shown to play a central role in cardiomyocyte death induced by multiple insults (12, 13, 24, 25), we postulate that CaMKII-δ9-mediated cardiomyocyte death may be involved in heart failure elicited by various pathological insults.

## Conclusion

In conclusion, we provided the evidence that CaMKII-δ9 mediates cardiomyocyte death, but not cardiac hypertrophy, to elicit cardiomyopathy and heart failure. Furthermore, CaMKII-δ9 promotes cardiomyocyte death and heart failure in both male and female animals. Our findings not only deepen our understanding of the role of CaMKII-δ9 in cardiac pathology but also provide new insights into the mechanisms and therapy of heart failure.

## Data Availability Statement

The original contributions presented in the study are included in the article/supplementary materials, further inquiries can be directed to the corresponding author/s.

## Ethics Statement

The animal study was reviewed and approved by the Institutional Animal Care and Use Committee of Peking University.

## Author Contributions

MZ, JZ, and YZ proposed the hypothesis, generated the initial idea, conducted key experiments and data analysis, and wrote the manuscript. WZha, QH, LJ, PX, WZhe, and HS researched data and contributed to discussion. All authors contributed to the article and approved the submitted version.

## Funding

This work was supported by the National Key R&D Program of China (2018YFA0800501), and the National Natural Science Foundation of China (92168114, 31671177, 81630008, 81790621, 81872752, and 31521062).

## Conflict of Interest

The authors declare that the research was conducted in the absence of any commercial or financial relationships that could be construed as a potential conflict of interest.

## Publisher's Note

All claims expressed in this article are solely those of the authors and do not necessarily represent those of their affiliated organizations, or those of the publisher, the editors and the reviewers. Any product that may be evaluated in this article, or claim that may be made by its manufacturer, is not guaranteed or endorsed by the publisher.
